# Lost in Cognition: Paraneoplastic Rhombencephalitis With Anti-Ri/Antineuronal Nuclear Antibody Type 2 (ANNA-2) Antibodies Presenting as Rapid-Onset Cognitive Decline and Ophthalmoplegia-Ataxia With Reversible Brainstem Lesion

**DOI:** 10.7759/cureus.91931

**Published:** 2025-09-09

**Authors:** Zeinab Awada, Ayman Alboudi, Iffat Jabeen, Edward Yu

**Affiliations:** 1 Neurology, Staten Island University Hospital, New York, USA; 2 Neurology, Wayne State University Detroit Medical Center, Detroit, USA

**Keywords:** anna-2 antibody, anti-ri antibodies, autoimmune encephalitis, early breast cancer, paraneoplastic encephalitis syndromes

## Abstract

Anti-Ri/antineuronal nuclear antibody type 2 (ANNA-2) autoantibodies have emerged as significant markers within the realm of paraneoplastic neurological syndromes (PNS), which are recognized for their connection with onconeural antibodies and their potential to cause neurological manifestations in the presence of an underlying malignancy. If left untreated, PNS involving anti-Ri antibodies can result in substantial morbidity and mortality, and the heterogeneous range of associated syndromes and clinical presentations often complicates the diagnosis. We report the case of a 67-year-old Asian female patient with a history of hypertension and diabetes mellitus who developed progressive gait ataxia, diplopia, dysphagia, facial twitching, and cognitive decline over three months. Neurological examination revealed conjugate horizontal gaze palsy, partial ptosis, oromandibular dystonia, hyperreflexia, and ataxia. Brain magnetic resonance imaging (MRI) showed non-enhancing T2/fluid-attenuated inversion recovery (FLAIR) patchy signal changes in the brainstem, and cerebrospinal fluid (CSF) analysis revealed mildly elevated protein and pleocytosis. ANNA-2 antibodies were positive in both serum and CSF, prompting a malignancy workup that uncovered an invasive, poorly differentiated ductal carcinoma of the breast. The patient was started on high-dose corticosteroids, resulting in partial radiological and clinical improvement. In conclusion, a subacute gait ataxia, ophthalmoparesis, and jaw dystonia should raise suspicion for anti-Ri/ANNA-2 rhombencephalitis, warranting prompt malignancy evaluation and early immunotherapy to optimize outcomes.

## Introduction

Antineuronal nuclear antibody type 2 (ANNA-2) encephalitis is a rare, autoimmune-mediated neurological disorder associated with the presence of anti-Ri/ANNA-2 antibodies in the patient's serum and/or cerebrospinal fluid (CSF) [1]. This paraneoplastic syndrome primarily affects the central nervous system, leading to a broad spectrum of neurological manifestations, including cerebellar ataxia, opsoclonus-myoclonus, and brainstem dysfunction [2]. The pathophysiology of ANNA-2-positive encephalitis involves an immune-mediated response directed against the nuclear Ri protein [3]. However, the precise mechanism of antibody-induced neuronal injury remains poorly understood, highlighting the need for further research in this area.

Given the rarity of this condition, diagnosis can be challenging and is often delayed, resulting in suboptimal patient outcomes. Early recognition and timely intervention are critical to improving prognosis. Furthermore, the established association of ANNA-2 antibodies with underlying malignancies, particularly small-cell lung carcinoma and gynecological tumors, necessitates thorough cancer screening in affected individuals [4].

This case report describes the clinical presentation, diagnostic evaluation, and management of a patient diagnosed with ANNA-2-positive encephalitis. We aim to raise awareness of this distinct neurological syndrome to promote earlier diagnosis and appropriate treatment, thereby improving overall patient care and outcomes.

## Case presentation

A 67-year-old Asian female patient with a previous medical history of controlled hypertension and diabetes mellitus presented with a three-month history of progressive gait instability, diplopia, dysphagia, facial twitching, and cognitive decline. The patient was unable to perform her activities of daily living, such as bathing, dressing, and preparing her food, but she was still able to eat without help. Neurological examination revealed conjugate horizontal gaze palsy with impaired horizontal gaze resulting in binocular diplopia, particularly on lateral gaze. Additionally, there was bilateral partial ptosis, while the evaluation of the other cranial nerves was normal. Motor examination demonstrated full strength in both upper and lower extremities. Repetitive, involuntary, spastic movements of the jaw and lower facial muscles were observed, consistent with oromandibular dystonia. Deep tendon reflexes were brisk (3+) in all extremities, and marked bilateral dysmetria was present on finger-to-nose and shin-to-heel testing. 

**Figure 1:**
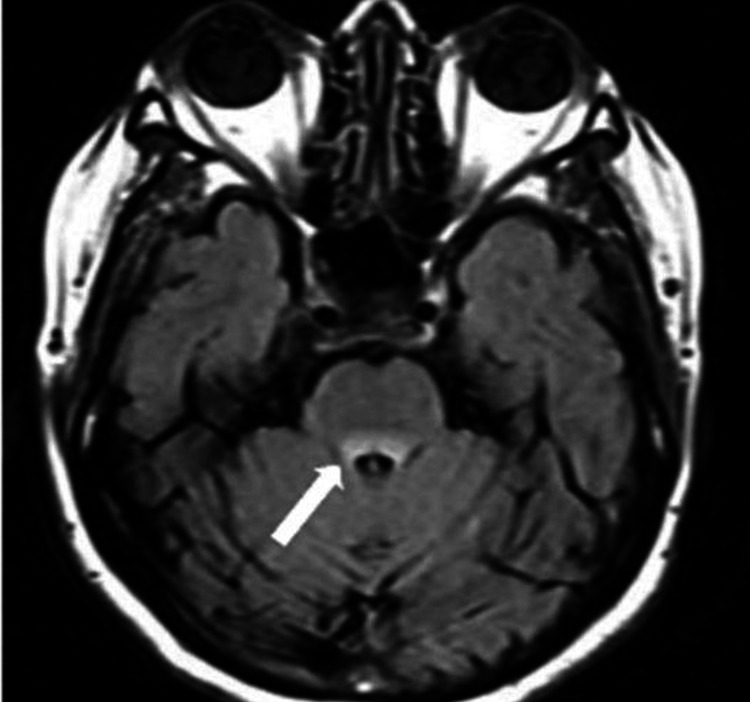
Axial view of brain MRI showing the mesencephalic tegmentum signal abnormality on FLAIR MRI: magnetic resonance imaging; FLAIR: fluid-attenuated inversion recovery

Magnetic resonance imaging (MRI) of the brain (3 Tesla, with and without contrast) demonstrated patchy T2-weighted fluid-attenuated inversion recovery (FLAIR) hyperintensities within the brainstem, without mass effect, diffusion restriction, or contrast enhancement. These signal abnormalities extended into the superior cervical spinal cord (Figure 1).

No significant abnormalities were seen elsewhere in the neuroaxis. An electroencephalogram (EEG) was done and did not show any seizures or epileptiform discharges during some of the facial movement episodes. Given the subacute progression of cognitive dysfunction, ophthalmoparesis, postural instability, bulbar symptoms, jaw dystonia, and hyperreflexia, combined with the imaging findings, a paraneoplastic syndrome was suspected. Empiric high-dose intravenous methylprednisolone 1000 mg daily was administered for five days, followed by prednisone 60 mg daily tapered by 10 mg every week, leading to the partial improvement of ophthalmoparesis.

Initial laboratory studies revealed an elevated erythrocyte sedimentation rate (ESR) of 90 mm/hr (reference: 0-20 mm/hr) and a mildly elevated C-reactive protein (CRP) of 7.5 mg/L (reference: 0-5 mg/L). Additional investigations, including vitamin levels (A, D, K, B12, B1, B6), heavy metal panel (that include mercury, lead, arsenic, cadmium, cobalt, and thallium), thyroid function (thyroid-stimulating hormone (TSH)), thyroid antibodies (thyroid peroxidase (TPO)), antinuclear antibody (ANA), double-stranded DNA (dsDNA), antineutrophil cytoplasmic antibodies (c-ANCA and p-ANCA), serum and urine electrophoresis, acetylcholine receptors antibodies and antiganglioside panels, voltage-gated calcium channel (VGCC), myelin oligodendrocyte (MOG), and neuromyelitis optica (NMO) antibodies, were unremarkable. CSF analysis revealed mildly elevated protein (46 mg/dL; reference: 15-45 mg/dL) and lymphocytic pleocytosis (9 cells/µL; reference: 0-5 cells/µL) (Table 1).

**Table 1:** Comprehensive lab work ESR: erythrocyte sedimentation rate; CRP: C-reactive protein; TSH: thyroid-stimulating hormone; TPO: thyroid peroxidase; ANA: antinuclear antibody; dsDNA: double-stranded DNA; c-ANCA: cytoplasmic antineutrophil cytoplasmic antibody; p-ANCA: perinuclear antineutrophil cytoplasmic antibody; VGCC: voltage-gated calcium channel; MOG: myelin oligodendrocyte glycoprotein; NMO: neuromyelitis optica; CSF: cerebrospinal fluid; ANNA-2: antineuronal nuclear antibody type 2

Test name	Result	Reference range
ESR	90 mm/hr	0-20 mm/hr
CRP	7.5 mg/L	0-5 mg/L
Vitamin A	Normal	Normal
Vitamin D	Normal	Normal
Vitamin K	Normal	Normal
Vitamin B12	Normal	Normal
Vitamin B1	Normal	Normal
Vitamin B6	Normal	Normal
Heavy metal panel	Negative	Negative
TSH	Normal	Normal
TPO antibodies	Negative	Negative
ANA	Negative	Negative
dsDNA	Negative	Negative
c-ANCA	Negative	Negative
p-ANCA	Negative	Negative
Serum protein electrophoresis	Normal	Normal
Urine protein electrophoresis	Normal	Normal
Myasthenia panel	Negative	Negative
Antiganglioside antibodies	Negative	Negative
VGCC	Negative	Negative
MOG	Negative	Negative
NMO antibodies	Negative	Negative
CSF protein	46 mg/dL	15-45 mg/dL
CSF cell count	9 cells/µL	0-5 cells/µL
ANNA-2, serum	Positive	Negative
ANNA-2, CSF	Positive	Negative

The autoimmune/paraneoplastic encephalopathy panel in serum (ENS2 immunofluorescence assay that includes amphiphysin, CRMP, DPPX, GRAP, mGluR1, IgLON5, ANNA-1, ANNA-2, ANNA-3, antiglial nuclear type 1, Purkinje cell cytoplasmic antibody, LGI1, CASPR2, GAD65, AMPA-R, GABA-B) was positive for ANNA-2. CSF testing confirmed the same antibody, supporting a diagnosis of ANNA-2-associated paraneoplastic encephalopathy. A computed tomography (CT) scan of the chest, abdomen, and pelvis was unremarkable.

**Figure 2:**
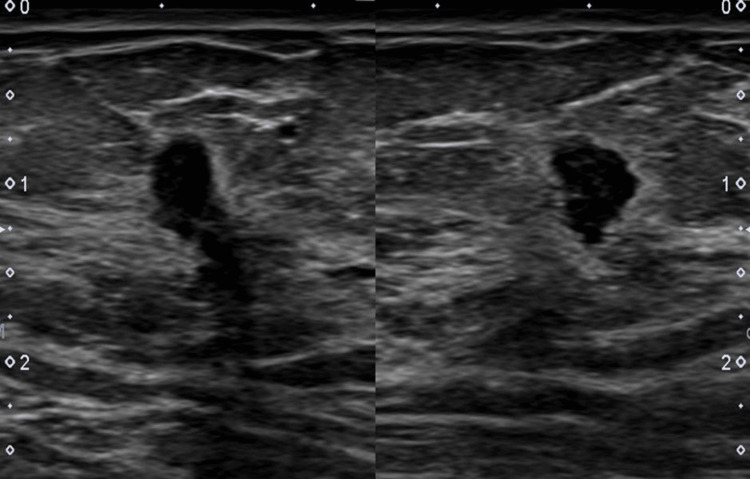
Ultrasound of the right breast showing an irregular hypoechoic mass at the 9-10 o'clock axis, 6 cm from the nipple, which measures 1.1×0.5×0.6 cm and at the 9 o'clock axis, 6 cm from the nipple, which measures 0.7×0.5×0.2 cm

Given the antibody profile, positron emission tomography (PET)/CT imaging was pursued, revealing a minimally fluorodeoxyglucose (FDG)-avid nodular density in the right breast. Follow-up mammography and targeted breast ultrasound confirmed the presence of two irregular hypoechoic masses (Figure 2).

Ultrasound-guided biopsy demonstrated invasive, poorly differentiated ductal carcinoma with anaplastic features, necrosis, lymphoplasmacytic inflammation, and scattered multinucleated tumor giant cells.

**Figure 3:**
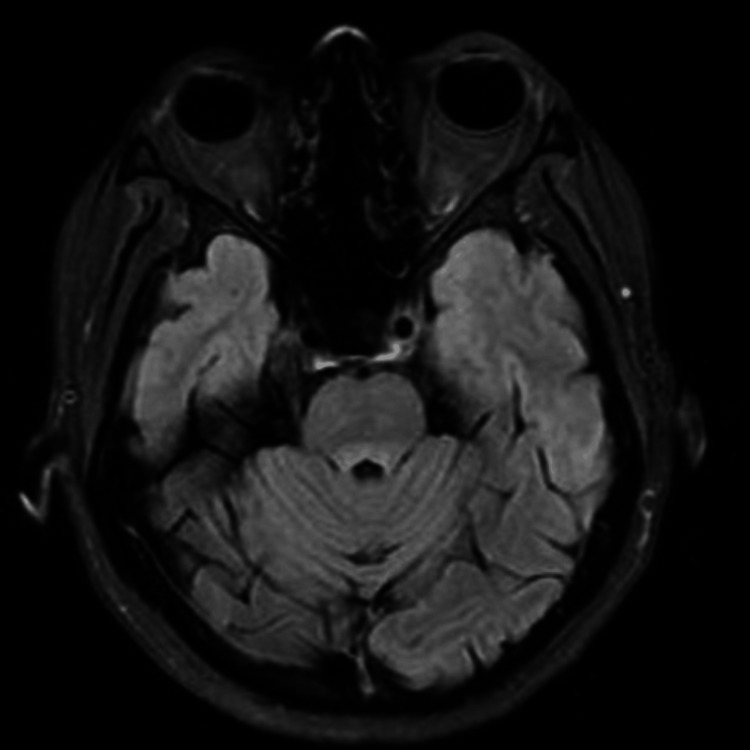
T2 FLAIR study of brain MRI showing the resolving signal abnormality in the dorsal pons MRI: magnetic resonance imaging; FLAIR: fluid-attenuated inversion recovery

A diagnosis of paraneoplastic brainstem encephalitis associated with ANNA-2 antibodies, secondary to breast cancer, was confirmed based on the clinical findings, antibody serology, and histopathology. Repeat brain MRI at four weeks demonstrated resolution of the dorsal pontine signal abnormalities (Figure 3), though clinical improvement was minimal. The patient remained on a maintenance corticosteroid regimen, with plans for continued follow-up in oncology.

## Discussion

Paraneoplastic neurological syndromes (PNS) encompass a heterogeneous array of complications linked to malignancy, arising from an immune response directed against the tumor that affects various parts of the nervous system. When this syndrome involves the brainstem, it manifests as paraneoplastic rhombencephalitis. In PNS, neoplasms aberrantly express antigens typically restricted to the nervous system, triggering an immune-mediated cross-reactive response [5]. The structural analogy shared between tumor-specific antigens and neuronal components provokes an immunological reaction in which antibodies directed against tumor antigens also cross-react with neural epitopes. The anti-Ri/ANNA-2 antibody is one such onconeural antibody, most commonly associated with breast carcinoma and small-cell lung carcinoma [5]. Other malignancies include fallopian tube, ovarian, bladder, and gastric neuroendocrine cell carcinomas, seminomas, and carcinoid tumors [6]. Neurological syndromes associated with ANNA-2 antibodies encompass brainstem syndromes (including opsoclonus, myoclonus, or both), cerebellar syndrome, myelopathy, peripheral neuropathy, cranial neuropathy, movement disorders, encephalopathy, Lambert-Eaton myasthenic syndrome, and seizures [7]. Jaw dystonia and laryngospasm are observed in approximately 19% of cases [6]. Diagnosis of ANNA-2 syndrome typically follows the current diagnostic criteria for PNS established by the International PNS-Care Panel. ANNA-2 syndrome involves neurologic dysfunction affecting multiple regions of the nervous system and is frequently associated with underlying malignancy. It is characterized by an immune-mediated pathogenesis supported by the detection of specific neuronal antibodies [1]. However, well-characterized onconeural antibodies are detected in only 25% of clinically selected patients [1]. Diagnostic evaluation includes a brain MRI, which may show T2 hyperintensities in the brainstem, with or without gadolinium enhancement. These lesions may be reversible with treatment and do not always correlate with clinical improvement [8]. Our patient showed radiological improvement in the brainstem lesion but minimal clinical recovery, consistent with reports [9]. ANNA-2 PNS shows a female predominance, with breast cancer being the most frequently associated malignancy. Importantly, among reported breast cancer cases, including ours, none exhibited human epidermal growth factor receptor 2 (HER2) expression [10]. In our case, the patient developed brainstem syndrome and rapidly progressive dementia within three months. Interestingly, routine mammography prior to symptom onset was unremarkable. Since PNS often precedes the diagnosis of the underlying tumor, comprehensive screening for occult malignancy is essential. In select cases, clinical and radiologic evaluations are supplemented with FDG-PET. In our patient, initial imaging studies were inconclusive, necessitating PET to identify a primary malignancy. When malignancy is not initially found, follow-up evaluations every six months for up to four years are advised. Approximately 80% of PNS cases are diagnosed with cancer within 4-6 months of symptom onset [1]. Response to immunotherapy in ANNA-2-associated PNS is often limited. However, early initiation of cancer-directed therapy and immunomodulation may stabilize disease progression. Management typically involves both oncologic treatment and immunotherapy, with intravenous methylprednisolone commonly used as first-line therapy.

Patients with anti-Ri (ANNA-2)-associated encephalitis often experience significant long-term neurological sequelae, primarily affecting the brainstem and cerebellum. Persistent deficits include disabling cranial neuropathies, chronic ataxia, and severe bulbar dysfunction, most notably jaw dystonia and recurrent laryngospasm. Jaw dystonia can markedly impair oral intake, leading to profound weight loss, malnutrition, and the need for enteral feeding. Recurrent laryngospasm, a distinct and potentially fatal manifestation, poses a serious risk of airway obstruction and has been directly implicated in mortality in several cases [11]. In a cohort of patients with ANNA-2 seropositivity, nearly 20% exhibited this phenotype, highlighting its relative frequency in this paraneoplastic syndrome [6].

Neuropathologic findings support an immune-mediated etiology, with CD8⁺ T-cell infiltration, axonal loss, and gliosis observed in the brainstem and descending tracts [6]. Despite aggressive immunotherapy, including corticosteroids, intravenous immunoglobulin, and cytotoxic agents, complete neurological recovery remains uncommon, and symptoms often progress despite treatment [6,7,11]. Chronic bulbar dysfunction and dysphagia frequently require tracheostomy or long-term nutritional support [6,7]. Additional long-term deficits include gaze palsies, gait instability, and spasticity, all of which contribute to enduring disability. Cognitive and psychiatric symptoms may also occur, particularly in patients with concurrent limbic involvement [11].

Importantly, prognosis is influenced by the underlying malignancy and the severity of the initial neurologic insult. Although tumor-directed therapy is essential, the neurologic outcome is often poor, with residual symptoms persisting in most patients despite treatment [7,11]. 

## Conclusions

Subacute gait ataxia, ophthalmoparesis, cognitive decline with bulbar manifestations, and jaw dystonia should raise suspicion for ANNA-2 rhombencephalitis. It represents complex and challenging neurological disorders, where the precise mechanisms underlying the development of this syndrome remain not clearly understood. Although subsequent imaging studies may exhibit improvement in signal abnormalities after treatment, radiological improvement does not necessarily translate into clinical improvement. Early recognition is important in facilitating prompt cancer detection, intervention, and, consequently, enhanced patient outcomes.

## References

[1] Freydl E, Tinchon A, Blauensteiner K, Oberndorfer S (2024). Anti-Ri paraneoplastic neurological syndrome presenting with bilateral cranial nerve VI palsy and jaw dystonia-a distinctive syndrome within the anti-Ri spectrum?. Wien Med Wochenschr.

[2] Peterson K, Rosenblum MK, Kotanides H, Posner JB (1992). Paraneoplastic cerebellar degeneration. I. A clinical analysis of 55 anti-Yo antibody-positive patients. Neurology.

[3] Graus F, Dalmou J, Reñé R (1997). Anti-Hu antibodies in patients with small-cell lung cancer: association with complete response to therapy and improved survival. J Clin Oncol.

[4] Knudsen A, Monstad SE, Dørum A (2006). Ri antibodies in patients with breast, ovarian or small cell lung cancer determined by a sensitive immunoprecipitation technique. Cancer Immunol Immunother.

[5] Boch M, Rinke A, Rexin P (2014). Paraneoplastic brainstem encephalitis in a patient with exceptionally long course of a metastasized neuroendocrine rectum neoplasm. BMC Cancer.

[6] Pittock SJ, Parisi JE, McKeon A (2010). Paraneoplastic jaw dystonia and laryngospasm with antineuronal nuclear autoantibody type 2 (anti-Ri). Arch Neurol.

[7] Pittock SJ, Lucchinetti CF, Lennon VA (2003). Anti-neuronal nuclear autoantibody type 2: paraneoplastic accompaniments. Ann Neurol.

[8] Kim H, Lim Y, Kim KK (2009). Anti-Ri-antibody-associated paraneoplastic syndrome in a man with breast cancer showing a reversible pontine lesion on MRI. J Clin Neurol.

[9] Alkabie S, Chang YC, Budhram A, Racosta JM (2022). Pearls & oy-sters: gait instability, jaw dystonia, and horizontal diplopia in a woman with anti-Ri antibodies and breast cancer. Neurology.

[10] Tisavipat N, Chang BK, Ali F (2023). Subacute horizontal diplopia, jaw dystonia, and laryngospasm. Neurol Neuroimmunol Neuroinflamm.

[11] Simard C, Vogrig A, Joubert B (2020). Clinical spectrum and diagnostic pitfalls of neurologic syndromes with Ri antibodies. Neurol Neuroimmunol Neuroinflamm.

